# AltTrans: Transcript pattern variants annotated for both alternative splicing and alternative polyadenylation

**DOI:** 10.1186/1471-2105-7-169

**Published:** 2006-03-23

**Authors:** Vincent Le Texier, Jean-Jack Riethoven, Vasudev Kumanduri, Chellappa Gopalakrishnan, Fabrice Lopez, Daniel Gautheret, Thangavel Alphonse Thanaraj

**Affiliations:** 1 European Bioinformatics Institute, Wellcome Trust Genome Campus, Hinxton, Cambridge, CB10 1SD, UK; 2 INSERM ERM206, Université de la Méditerranée, Luminy case 928 – 13 288 Marseille Cedex 09, France; 3 18 Crispin Close, Haverhill, Suffolk, CB9 9PT, UK; 4 4 Copperfields, Saffron Walden, Essex, CB11 4FG, UK

## Abstract

**Background:**

The three major mechanisms that regulate transcript formation involve the selection of alternative sites for transcription start (TS), splicing, and polyadenylation. Currently there are efforts that collect data & annotation individually for each of these variants. It is important to take an integrated view of these data sets and to derive a data set of alternate transcripts along with consolidated annotation. We have been developing in the past computational pipelines that generate value-added data at genome-scale on individual variant types; these include AltSplice on splicing and AltPAS on polyadenylation. We now extend these pipelines and integrate the resultant data sets to facilitate an integrated view of the contributions from splicing and polyadenylation in the formation of transcript variants.

**Description:**

The AltSplice pipeline examines gene-transcript alignments and delineates alternative splice events and splice patterns; this pipeline is extended as AltTrans to delineate isoform transcript patterns for each of which both introns/exons and 'terminating' polyA site are delineated; EST/mRNA sequences that qualify the transcript pattern confirm both the underlying splicing and polyadenylation. The AltPAS pipeline examines gene-transcript alignments and delineates all potential polyA sites irrespective of underlying splicing patterns. Resultant polyA sites from both AltTrans and AltPAS are merged. The generated database reports data on alternative splicing, alternative polyadenylation and the resultant alternate transcript patterns; the basal data is annotated for various biological features. The data (named as integrated AltTrans data) generated for both the organisms of human and mouse is made available through the Alternate Transcript Diversity web site at .

**Conclusion:**

The reported data set presents alternate transcript patterns that are annotated for both alternative splicing and alternative polyadenylation. Results based on current transcriptome data indicate that the contribution of alternative splicing is larger than that of alternative polyadenylation.

## Background

The three major regulatory mechanisms that bring about formation of alternative transcript patterns from an expressed gene act at the choice of alternative sites for transcription start (TS), splicing, and polyadenylation [1-4]. Use of alternative TS site and/or alternative polyA site often accompanies alternative splicing [1,4,5]. Currently there are efforts that collect data & annotation either for TS variants [1,6-9], or for splice variants [10-24], or for polyA variants [3,25-28]. These data sets provide a wealth of value-added annotation (such as tissue specificity, evolutionary conservation, and regulatory motifs). Given that there is a coupling between the machineries responsible for transcription initiation, splicing, and polyadenylation [29-33], it is important to take a coherent & integrated view of these individual variants and to derive a data set of alternate transcript patterns along with consolidated annotation. Previous attempts to integrate variants of splicing and polyadenylation (as well as of transcript start sites) introduced new methods for transcript assembly – notable examples being that of Kim et al [17], Sharov et al [34], and that of Zavolan et al [4]. General conclusions from these studies were that (i) a majority of transcription units showing multiple splice forms contain transcripts in which the apparent use of an alternative transcription start (or stop) is accompanied by alternative splicing of the initial (or terminal) exon; and that (ii) alternative splicing is a major contributor to transcriptome diversity.

We have been generating data sets on individual variant types; such data sets include AltSplice [11] and AltPAS [25]. Both the AltSplice and AltPAS pipelines generate genome-wide data based on Ensembl [35] gene annotation. The AltSplice pipeline examines gene-transcript alignments and delineates alternate splice events and alternate splice patterns; the pipeline characterises the generated data for various biological features. In the current work, we extend the AltSplice pipeline as AltTrans to annotate the observed splice patterns for terminating polyA site; the information on the polyA site for a splice pattern is derived by examining the transcript sequences that confirm the splice pattern; a splice pattern that could be annotated for terminating polyA site is termed as transcript pattern. The AltPAS pipeline examines gene-transcript alignments and identifies potential polyA sites independently of the underlying splicing patterns; as a result for a given set of genes, AltPAS derives a larger data set of polyA sites as compared to AltTrans.

The transcript patterns as derived by the AltTrans pipeline and the combined list of polyA sites as generated by AltTrans and AltPAS pipelines form the core of the data presented in this work; these basal data are annotated for various biological features. The resulting data for human and mouse is presented to the community in two forms: (i) through an FTP server as flat file distributions; and (ii) through user-friendly web query interfaces. Included in the database are those genes for which at least one transcript pattern (annotated both for splicing and terminating polyA site) was determined.

## Construction and content

The different pipelines discussed below are (i) AltSplice (that delineates splice related data namely, splice sites, splice patterns and splice events), (ii) AltTrans (that delineates transcript patterns along with annotation for terminating polyA site from AltSplice splice patterns, and (iii) AltPAS (that delineates all potential polyA sites on the gene independently of the underlying splicing patterns). Also discussed are the approaches to integrate the resulting data.

### Generating splice patterns: the AltSplice pipeline

The methodologies behind the AltSplice pipeline have been previously reported [11-13] and hence are briefly mentioned here. The considered gene set is that of the Ensembl genome annotation project [35]. For each of the considered genes, the nucleotide sequence of a region that extends Ensembl-defined gene boundaries by 3000 bases on either *flanking *side is extracted as AltSplice gene. Transcript (EST and mRNA) sequences as extracted from EMBL database [36] are used to generate a high quality data set of gene-transcript alignments showing more than one high scoring match between gene and transcript sequences. Any transcript sequence that aligns exclusively to the *flanking *regions of a gene is discarded. Further, any transcript sequence that aligns with more than one gene or ambiguously with more than one region on a single gene is discarded. Alignment gaps on gene sequences are considered as potential introns and their validation as transcript-confirmed introns is a crucial step in AltSplice pipeline (see [11-13] for validation procedures). Alignment matches on the gene sequence are accepted as a confirmed exon if flanked on either side by a confirmed intron. Thus each transcript sequences that maps to a gene is described by its exon-intron structure. Transcript sequences that map to a gene are then grouped into classes in a manner that the member transcripts from a class show same exon-intron structure. Longest representative from each such class denotes a unique splice pattern for the gene. Overlapping exons and introns from the isoform splice patterns are then examined to delineate alternative splice events.

### Redefinition of AltSplice gene region

An AltSplice gene represents a genomic region containing (5' flanking 3000 bases + Ensembl gene region + 3' flanking 3000 bases). Once the splice patterns are identified, we redefine the AltSplice gene region as below:

(i) If no splice pattern is observed extending into flanking regions, then AltSplice gene is trimmed down to that as annotated in Ensembl.

(ii) If a splice pattern is seen as extending into the 5' flanking region, the genome start location of the splice pattern forms the 5' bound for AltSplice gene.

(iii) If a splice pattern is seen as extending into the 3' flanking region, the genome end location of the splice pattern forms the 3' bound of AltSplice gene.

### Generating transcript patterns and 'terminating' polyA sites from AltSplice splice patterns: the AltTrans pipeline

**Figure 1:**
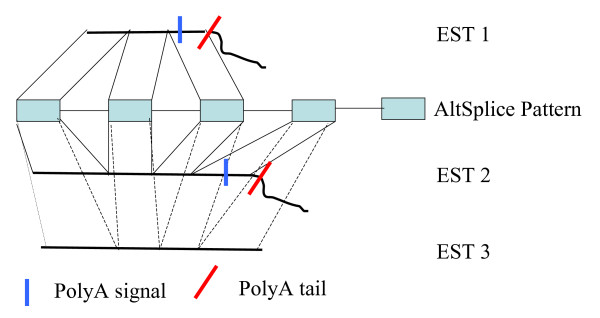
Derivation of transcript patterns by the AltTrans pipeline from AltSplice splice patterns. Each of the gene-transcript alignments from AltSplice is examined for the following: (i) the alignment shows a 3' dangling end on the EST/mRNA; (ii) such a dangling end shows a polyA tail sequence; and (iii) a polyA signal is seen on the gene within a maximum distance of 40 nts 5' to the cleavage position. Transcripts that show these features are grouped in a manner that each class of transcripts possesses the same exon/intron organisation and the same terminating polyA site. Such derived alternate transcript patterns are described as AltTrans transcript patterns. Note: Of the three EST's, that are all grouped under one AltSplice splice pattern, the EST3 does not show a "dangling end" and hence it is not considered further in the construction of AltTrans Transcript Patterns. EST1 and EST2 form two distinct transcript patterns that differ in terminating polyA sites.

AltSplice splice patterns and the gene-transcript alignments form the basis of AltTrans pipeline that delineates alternate transcript patterns (see Fig. 1). Each of the gene-transcript alignments confirming a splice pattern from AltSplice is examined for the presence of a polyA site that terminates the transcript sequence. Transcripts displaying a terminating polyA site are grouped in a manner that each class of transcript shares the same exon-intron structure and the same terminating polyA site; representative from each such class is termed a transcript pattern. These different steps are as detailed below.

*Detecting polyA sites from transcript sequences that confirm AltSplice patterns*. This procedure is of the following three steps:

#### (i) Detecting polyA tail and polyA cleavage (PAC) site

Each of the gene-transcript alignments is examined for the presence of a 3' dangling end on the transcript sequence. Only those alignments that show 3' dangling ends of length at least 8 bases are considered further. The transcript region -5 to +5 from the end of alignment is examined for the start of a polyA tail. A polyA tail is defined as a string of 8 or more adenosines. It is observed that a higher proportion of the dangling ends are short (5 to 50 bases) and often involve runs of adenosines. While it is possible that mRNAs can possess long polyA tails, we are worried about longer dangling ends since their extra lengths can be results of artefacts in EST sequences or of genomic 'contaminations' at the 3' ends of the ESTs. We do want to include transcript patterns involving those gene-EST alignments with long dangling ends; however, we want to be sure that such dangling ends contain genuine polyA tails and hence we tightened the requirements for polyA tail on long dangling ends as below: (length of dangling end : minimum length of polyA tail) as (= 50 : 8); (>50 & = 100 : 10); (>100 & = 150 : 15); and (>150 : 20). Since it is often the case that a run of adenosines is interrupted by non-adenosine bases, we allow mismatches at up to a maximum of 10% of the positions in the identified string provided the string still contains the required number of adenosines (as per the specification mentioned above); for this purpose, we increase the search window sequence in advance by 10% to take any mismatch into account. If more than one polyA tail is identified starting in the -5 to +5 region, the one with the highest composition of adenosines is chosen as the authentic polyA tail. The gene position corresponding to the start of polyA tail is considered as the cleavage site. As many as 75% of instances of dangling ends showing a putative polyA tail are of shorter lengths (<= 50 bases), 8% are of length 50–100 bases; 4% are of length 100–150 bases, and 13% are of length > 200 bases.

#### (ii) Detecting polyA signal (PAS)

A region on the gene sequence that aligns to the 40 nt transcript region 5' to the identified cleavage site is scanned for the presence of one of the 13 variant signals (namely, AAUAAA, AUUAAA, UAUAAA, AGUAAA, AAGAAA, AAUAUA, AAUACA, CAUAAA, GAUAAA, AAUGAA, UUUAAA, ACUAAA and AAUAGA) reported in the literature [3] with the criteria that no mismatch is allowed. For every gene-transcript alignment, all such motifs are identified. Of multiple matches, a representative motif is chosen as per the following criteria: (i) one that occurs within the region of -25 to -15 to the cleavage site is chosen; if multiple such motifs are seen within this region, the one of highest ranking (as ordered in [3] is chosen; of the highest ranking ones, the one that is close to the position of -20 is chosen; (ii) if no signal is seen in the -25 to -15 region, then those identified outside this region are examined; if multiple signals occur outside the region, the one of highest ranking located close to the position -20 is chosen. A higher proportion of gene-transcript alignments with longer dangling ends (that passed the test for presence of polyA tail) gets removed at this step, when compared to those with shorter dangling ends – we observe the following relationship between length of dangling end and proportion of transcripts failing the signal motif test: < 50 bases **: **9%; *>50 bases & = 100 bases ****:****14%; >100 bases & = 200 bases ****:****25%; and >200 bases : 33%*.

#### (iii) Grouping nearby cleavage sites and choosing a representative cleavage site as polyA site)

At this stage, gene-transcript alignments that do not show both a cleavage site and a polyadenylation signal are not considered further. Steps discussed so far identify for every gene a set of cleavage sites along with polyA signals. It is often the case that some of the identified cleavage sites are close to one another. Given that a polyA site can harbour multiple cleavage sites [3,25,37] and also that errors in sequences can lead to small differences in the locations of identified cleavage sites, it is possible that the adjacent sites are not distinct polyA sites. Thus it is essential to have a method in place to identify which of the close by sites can be chosen as an authentic polyA site. The identified cleavage sites are classified onto groups such that a member of a group differs from its immediate 5' neighbour by a maximum of 5 bases; the 5' most site from each such group is then chosen as the representative polyA site for that group of transcripts. Each member gene-transcript alignment of the group is annotated by such a representative polyA site and its associated signal motif.

#### Forming the transcript pattern classes

At this stage, for every gene a set of gene-transcript sequences with known exon-intron structure and terminating polyA site is available. The transcript sequences are then grouped into classes in such a manner that members of a class show same exon-intron structure and same terminal polyA site. The longest representative member of each such class is termed as a "Transcript Pattern".

### Identifying all potential polyA sites (independent of underlying splicing patterns): the AltPAS pipeline

The list of polyA sites as identified by AltTrans pipeline is by no means comprehensive for the reason that AltTrans examines only those gene-transcript alignments confirming AltSplice splice patterns. AltPAS is a generic pipeline that identifies all potential polyA sites irrespective of whether the examined gene-transcript alignments reveal any underlying splicing pattern or not. The methodology used is described in [25] and is briefly discussed below.

EST sequences and full-length cDNA sequences are obtained from transcript resources such as dbEST [38], H-Inv [39], and FANTOM [40]. Of the EST sequences, only those that are annotated as from the 3' end of gene are retained. Trailing polyA or polyT sequences of 5 nt or more are removed from the EST sequences. Both the 3' EST and cDNA sequences (termed transcript sequences) are aligned to the repeat-masked genome using the MegaBlast program [41]. High scoring matches are retained for further analysis. The matches are then clustered in a manner that transcript members from a cluster have their end positions located within a range of 10 nucleotides from each other. Each cluster is then analyzed using a sliding 10-nt window to locate the most likely cleavage site, defined as the position where the window contains the ends of most transcripts. Alignment hits with more than 5 unmatched positions at cleavage site are discarded. Cleavage sites that are flanked by A-rich region (at least 9 out of 10 nt positions are adenosines) in the 50 nt downstream genomic sequence, and those that do not contain one of the known polyA signals in the 30 nt upstream region are discarded. Of the remaining cleavage sites, only those that are supported by at least two transcript sequences are retained as potential polyA sites. The polyA sites, thus identified, are denoted using genome coordinates. Assignment of the detected polyA sites to Ensembl genes is carried out as below: A polyA site is assigned to the Ensembl gene to which the site's genome location can be mapped; if the genome location of a polyA site does not map to any annotated gene, it is assigned to the nearest 5' gene, provided that the distance to gene is less than 3000 bases.

### Integrating polyA sites from the AltPAS and AltTrans pipelines

Of the polyA sites identified by the AltPAS pipeline, considered further are only those that can be mapped within the bounds of AltTrans genes. PolyA sites identified by both pipelines are merged. Adjacent polyA sites are then grouped and a representative polyA site is chosen from each group. The procedure adopted for the grouping process is same as that used for grouping AltTrans polyA sites (discussed in earlier sections) with the following variation. If a group contains sites from both AltTrans and AltPAS, the 5' most AltTrans site is chosen as the representative. Such a set of representative polyA sites is subsequently used to annotate a gene with all potential polyA sites, and to annotate a transcript pattern for potentially "skipped" polyA sites. It is to be noted that a transcript pattern in the data set always ends with an AltTrans polyA site as the terminating polyA site.

## Discussion

**Figure 2:**
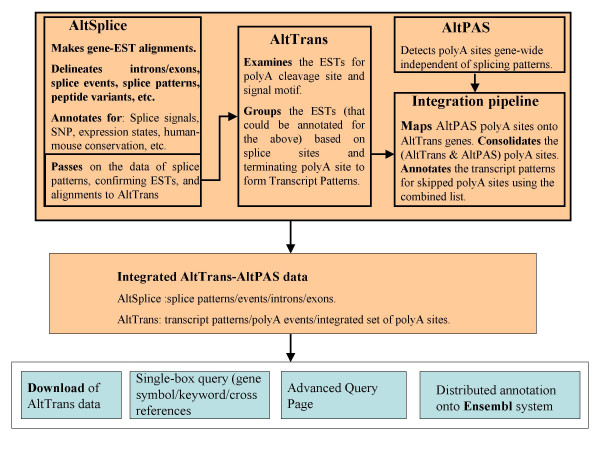
Illustration of the relationship between the AltTrans, AltSplice, and AltPAS pipelines/data.

We have been providing to the community an alternative splicing database (ASD) that integrates data from a computational pipeline (AltSplice) and from a manual curation effort (AEdb); such a database specializes on splicing events and their characteristics (see [11,12]). AltTrans, described in this manuscript, is an extension of AltSplice. It considers the splicing patterns as detected by AltSplice and examines the transcript sequences (that confirm each of the splicing patterns) for polyA sites. The transcript sequences thus annotated for both splicing pattern and polyA site are regrouped to form distinct transcript patterns. PolyA sites detected by AltPAS, an independent computational procedure, are also mapped to the AltTrans genes set. Transcripts produced by the AltTrans pipeline are presented as part of the ATD (Alternate Transcript Diversity) database. Figure 2 illustrates the relationship between AltSplice, AltTrans, and AltPAS pipelines/data. AltTrans is an important resource that elucidates the transcript complexity owing to alternative splicing and alternative polyadenylation. AltTrans will be further extended in future to provide information on transcript start as well. The possible applications include derivation of SAGE tags, derivation of exon junction probes for splice arrays, and primers for transcript-specific RT-PCR experiments.

Data sets of transcript patterns were derived for both human and mouse. Statistics on the generated data is presented in Table 1 and is discussed in the following sections.

### Human data set

The data set of human transcript patterns contains 7669 gene entries for each of which is derived at least one pattern that is fully annotated for both splicing and terminating polyA site. The total number of transcript patterns is 12559 (at an average of 1.6 per gene) encoded by 10221 terminating polyA sites. In 3179 of the 7669 AltTrans genes, two or more alternate transcript patterns could be observed. Inclusion of AltPAS polyA sites annotated an additional 6883 polyA sites raising the number of polyA sites mapped to 17104.

### Mouse data set

The data set of mouse transcript patterns contains 5862 gene entries. The total number of transcript patterns is 7755 (at an average of 1.3 per gene) encoded by 6976 terminating polyA sites. In 1548 of the 5862 AltTrans genes, two or more alternate transcript patterns could be observed. Inclusion of AltPAS polyA sites annotated an additional 2475 polyA sites raising the number of polyA sites mapped to 9451.

### Extent of alternative splicing versus alternative polyadenylation

Examination of data presented in Table 1 indicates that the proportion of human and mouse genes undergoing alternative splicing (at 74% and 65%, respectively) is higher than the proportion of genes undergoing alternative polyadenylation (at 60% and 42%, respectively). The above estimates are in agreement with those reported in literature – see [42] for estimate on alternative splicing and [3,25] on alternative polyadenylation. It is also seen that one in two human genes (close to one in three mouse genes) may undergo both alternative splicing and alternative polyadenylation. Considering only the polyA sites detected by the AltTrans pipeline (which requires that transcripts that confirm a polyA site also confirm the splicing of the transcript pattern) reveals a conservative estimate for extent of alternative polyadenylation at 27% for human and 18% for mouse.

### Limitations with regard to low number of reported transcript patterns

There is a large discrepancy in the numbers for observed splice patterns and observed transcript patterns. While the average number of observed splice patterns per human gene is 5.4, the average number of observed transcript patterns is a mere 1.6 (the corresponding numbers in the case of mouse data are 4.6 and 1.3). This discrepancy is due to the fact that for an EST/mRNA sequence to confirm a transcript pattern, it is required that the sequence confirms both the splicing and terminating polyA site. EST sequences do not often cover simultaneously both the internal and 3' regions of the gene – this is reflected in the observed numbers (see Table 1) for the EST/mRNA sequences that confirm splice patterns and transcript patterns (e.g. of the 837828 EST/mRNA sequences that confirm the human splice patterns, a mere 38731 contain enough information to confirm transcript pattern). As a result, the number of identified polyA sites by the AltTrans pipeline is expected to be reduced AltTrans detected 10221 polyA sites in 7669 human genes. AltPAS, that detects polyA sites independently of the underlying splicing process, mapped a further 6883 polyA sites to the same set of 7669 human genes. It is possible to increase the number of transcript patterns by using the AltPAS polyA sites as well to annotate the gene-transcript alignment for 'terminating' polyA site; however, we have restrained from doing this for the reason that it is our intention to provide a high quality set of transcript patterns, individual structural elements of which are confirmed by same set of EST/mRNA sequences.

### Heterogeneity of cleavage sites

**Figure 3:**
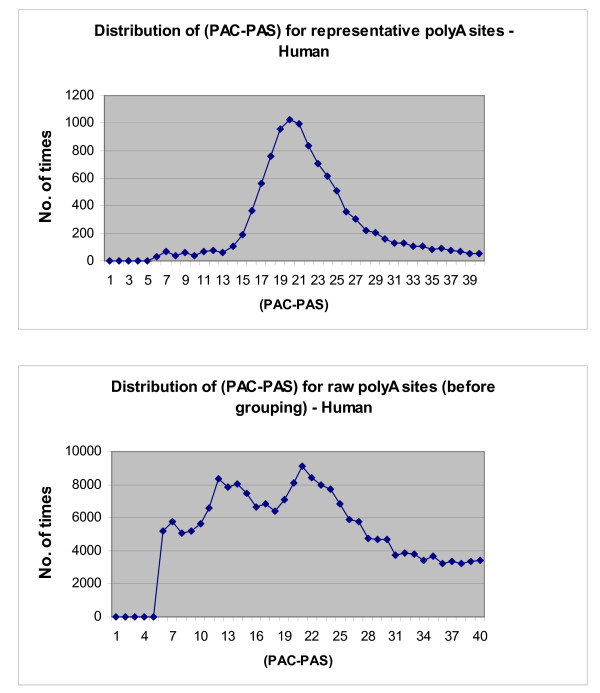
Distribution of spacing between polyA cleavage (PAC) site and polyA signal (PAS) in human transcript patterns from AltTrans. The bottom inset uses the data set of heterogeneous polyA sites; the top inset uses the data set of representative polyA sites (Nearby heterogeneous polyA sites are grouped and a representative polyA site is chosen – see text for methods).

It is known that a polyA site can harbour multiple cleavage sites and that polyadenylation can be an imperfect process [3,25,37,43]. However, it is possible that the small differences in the locations of multiple cleavage sites can be due to artefacts in EST sequences. The method that we adopted to select a polyA site from multiple cleavage sites involve grouping the sites in a manner that each member of the group differ from its 5' neighbour by a maximum of 5 bases. Upon examination of the distance between the locations of the 5'-most and 3'-most member sites in every group, it is seen that such an 'inner group distance' is non-zero only in 25% instances of the observed groups. An inner group distance of = 5 bases is seen only in 8% of instances. It may be possible that the grouping process can be refined further. However, it is often seen that the member cleavage sites of a group have the signal motifs identified at same gene position; and that the distribution of distance between the representative cleavage site and polyA signal (Fig. 3) show the expected normal distribution (while the distribution for the raw members show a bi-modal distribution). It is possible that the 3' ends of mRNAs are marked, in addition to polyA signal and polyA tail, by regions with distinct nucleotide compositional biases [43]. It is expected that incorporation of such signatures and statistical approaches (such as the one implemented in [43]) will lead to improvements in our above-discussed methods.

### Core data and derived annotations

The core of the generated data comprises the following components: (i) Genes and transcript data; (ii) introns/exons, polyA sites; (iii) isoform splice patterns, isoform transcript patterns, isoform peptide sequences; and (iii) alternative splice events, alternative polyadenylation events. Various value-added annotations are also generated, some of which are as described below.

#### Preservation of splice events across species

An important part of our pipeline is to generate evolutionary profiles of gene expression patterns. Methods based on the identification of conserved introns/exons and of conserved splice events [44] have been standardized to delineate pairs of human and mouse genes that are orthologous to each other. This data enables studies on evolutionary profiles of expression patterns.

#### Association with data on genetic variation (SNP, single nucleotide polymorphism)

We have developed methods (as documented in the ATD web pages) to delineate the allele specificity of observed alternative splice patterns.

#### Derivation of peptide sequences coded by isoform splices patterns

We have developed methods to delineate the amino acid sequence of the protein sequences encoded by the isoform splice patterns.

### Data access and query interfaces

The data was generated as part of the European Project on Alternate Transcript Diversity (ATD). The data can be downloaded as flat files or queried through web interfaces. The web interface provides single-box query (where a user can search the database against a keyword or gene symbol or database cross-references) or a detailed query page that searches simultaneously both the human and mouse data or a query page that provides advanced searches to either human or mouse data.

Genes can be queried by chromosomal location, gene names and synonyms, protein keywords, and database cross-references [such as EMBL and UniProt accession numbers [36,45], HUGO gene symbols [46], Gene Ontology identifiers [47] and protein identifiers], types of splice events, types of polyA signal, number of observed polyA sites, and types of variations among isoform transcript patterns (a pair of isoform transcript patterns may differ only in splicing or only in polyadenylation or in both). Queries can be selectively restricted to specific sets of gene entries, such as set of human-mouse orthologous gene pairs or set of gene entries for which data on isoform peptide sequences is available.

### Data presentation (textual & graphical displays) and integration

An output page resulting from a query to the database lists for every gene entry all the available database cross-references; an important aspect being hyperlinks to orthologous genes from other organisms (currently implemented for human and mouse).

**Figure 4:**
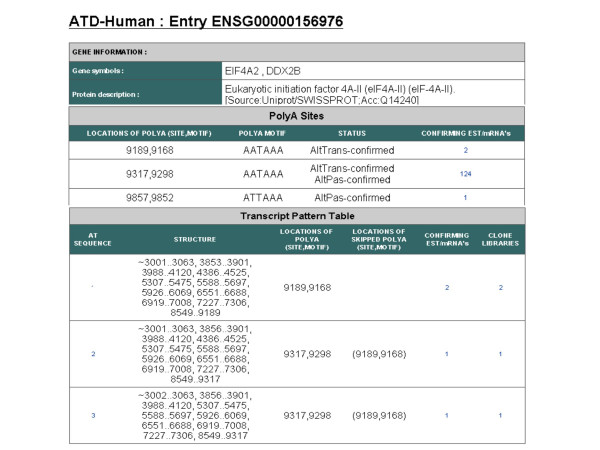
Examples of PolyA table and transcript pattern table. Locations of the polyA site and signal are as on the gene. Status of the polyA site refers to whether the site is identified by the AltTrans or AltPAS pipeline. Entry in the last column is hyperlinked to pages listing detailed information on the confirming transcript sequences.

Observed PolyA sites and transcript patterns are presented in tabular forms (Fig. 4). Typical information on a polyA site includes gene locations of the cleavage site and of the polyA signal, the signal sequence, and hyperlink to a page that lists the EST/mRNA sequences that confirm the polyA site. Typical information on a transcript pattern includes exon-intron structure of the pattern, locations of the 'terminating' polyA site (along with that of the polyA signal), polyA sites that are skipped in the formation of the pattern, hyperlinks to pages that list EST/mRNA sequences that confirm the pattern.

**Figure 5:**
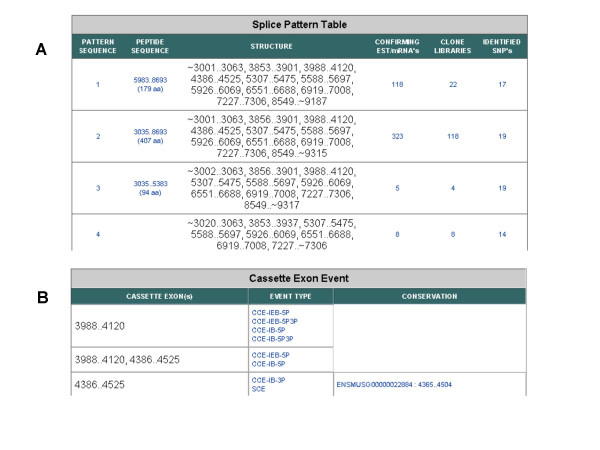
Example of splice pattern table and splice event table. Locations of exons are as on the gene.**Inset A: **Entry in column 1 is hyperlinked to a page listing the sequence of the splice pattern. Entry in column 2 gives the coding start & end positions on the gene and the length of the translated peptide sequence and is hyperlinked to a page listing the peptide sequence. Entry in column 3 lists the structure of the splice pattern as a string of exons. Entry in column 4 is hyperlinked to pages listing detailed information on the confirming transcript sequences. Entry in column 5 is hyperlinked to pages listing EST/mRNA sequences. Entry in column 6 is hyperlinked to pages listing allele specificity of the splice pattern. **Inset B: **Column 1 lists the exons involved in the event (in this example cassette exon event). Column 2 indicates whether the event involves modifications in the flanking exons as well; entries are hyperlinked to pages listing detailed information on the event. Column 3 indicates the identifier of the orthologous gene and the coordinates of the exon orthologous to the one presented in column1; the entry is hyperlinked to the orthologous gene entry.

Observed introns & exons are listed, and are hyperlinked to a page presenting data on EST/mRNA sequences that confirm these features. Observed splice patterns and events are listed (Fig. 5) and are hyperlinked to pages that list information on confirming EST/mRNA sequences. Typical information on a splice pattern includes hyperlinks to pages listing the coding information & sequences of the isoform peptides, detailed exon-intron structures & sequences of the isoform splice sequences, or listing the observed SNP positions and allele specificity. Typical information on a splice event includes information on the type of event, exon/intron feature that undergoes alternative splicing, hyperlink to a page giving details on the exon/intron features involved in the alternative splicing, or hyperlinks to the event in an orthologous gene from another species.

**Figure 6:**
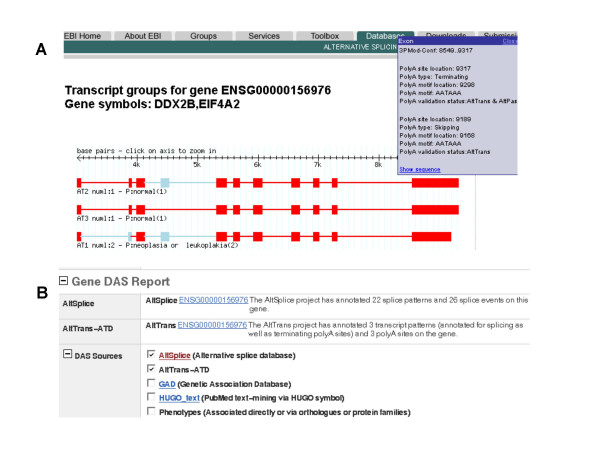
**Inset A: **Example of transcript pattern view. Exons are indicated by boxes and introns by lines. Exons/introns that are variants are indicated in blue colour. Browsing the cursor over various elements of a pattern displays pop-up's giving detailed information on the elements. The displayed pop-up in this example shows information on the polyA sites that maps to the alternate transcript pattern AT2; of these two polyA sites, the first one (located at gene position 9317) terminates the transcript pattern while the second one (located at gene position 9189) is skipped and is not used as a terminating polyA in the formation of this pattern. **Inset B: **Example snapshot of a portion of Ensembl gene display page to illustrate the integration of the AltTrans data in Ensembl genome annotation project.

Pattern viewers that give visual presentation of the observed isoform splice pattern structures and of the observed transcript pattern structures are provided. An example of transcript pattern view is presented in Fig. 6A. Each element of the pattern such as exon/intron/polyA site and the pattern as such is hyperlinked to pages giving detailed information (including nucleotide sequence, and detected signals).

The AltTrans data has been integrated with the Ensembl genome annotation project and is visible as DAS (Distributed Annotation System) tracks from the gene view and contigview pages in Ensembl genome browser (Fig. 6B).

## Conclusion

We present here an integrated data set of transcript-confirmed introns/exons, polyA sites, isoform splice patterns, isoform transcript sequences, isoform peptide sequences, alternative splice events, and alternative polyadenylation events. The data is presented for both mouse and human. Future work will aim to annotate the alternate transcripts for transcription start sites and their variants. In its future extension, this work should ultimately present high quality data on full-length transcript patterns annotated for transcription start site, splice sites, and polyadenylation sites; with each of these individual signals annotated for variations and for biological characteristics such as regulatory motifs and evolutionary profile.

## Availability and requirements

Release 1 of the integrated AltTrans data, presented in this manuscript, is available from . Enquiries on accessing the data can be mailed to asd-ebi@ebi.ac.uk.

## Abbreviations

TS: transcription start; FTP: file transfer protocol; EST: expressed sequence tag; mRNA: messenger RNA; cDNA – copy DNA; polyA: polyadenylation; PAC: polyadenylation cleavage; PAS: polyadenylation signal; dbEST: database of Expressed Sequence Tags; H-Inv: Human-Invitational Database; FANTOM: Functional Annotation of the mouse; BLAST: Basic Local Alignment Search Tool; ASD: Alternative Splicing Database; AEdb Alternative Exon Database; ATD: Alternate Transcript Diversity Database; SAGE: Serial Analysis of Gene Expression; RT-PCR: reverse transcription-polymerase chain reaction; UniProt: Universal Protein Resource.

## Authors' contributions

DG is responsible for formulating the AltPAS pipeline. TAT is responsible for formulating and developing the methods for the   AltTrans, AltSplice, the data integration pipelines, the annotation   modules, and the database & query interfaces. TAT has written the manuscript and DG has contributed to the drafting process. TAT headed the team at EBI. VLT has developed the software code for the database & interfaces and   for the annotation module of SNP-mediated splicing. JR has developed the software code for the AltTrans, AltSplice and the data integration pipelines. VK has developed the software code for the module of human-mouse conservation. CG has developed the software code for the module of deriving data on protein isoforms. FL has developed the software code for the AltPAS pipeline.

## Figures and Tables

**Table 1:** Statistics on Transcript Pattern Variant Data presented in this work.

	Human	Mouse
Genes	7669	5862
Splice patterns (& average number per gene)	41201 (5.4)	27132 (4.6)
Transcript patterns (& average number per gene)	12559 (1.6)	7755 (1.3)
		
PolyA sites as detected by AltTrans pipeline^1 ^(& average number per gene)	10221 (1.3)	6976 (1.19)
AltTrans polyA sites that are "skipped"	2468	1113
ATD PolyA sites as detected by AltTrans and AltPAS^2 ^pipelines (& average number per gene)	17104 (2.2)	9451 (1.61)
ATD polyA sites that are "skipped"	5459	2214
Genes showing splice events (as seen among the splice patterns)	5672 **(74%)**	3825 **(65%)**
Genes showing multiple polyA sites (considering the polyA sites from both AltTrans and AltPAS)^3^	4603 **(60%)**	2456 **(42%)**
Genes showing both splice events and multiple polyA sites (considering the polyA sites from both AltTrans and AltPAS)	3523 (46%)	1718 (29%)
Genes showing multiple polyA sites (considering only the AltTrans polyA sites)^4^	2053 **(27%)**	1026 **(18%)**
Genes showing both splice events and polyA events (considering only the AltTrans polyA sites)	1679 (22%)	736 (13%)
		
Genes showing >= 2 Splice Patterns	6859	4989
Genes showing >= 2 Transcript patterns	3179	1548
EST/mRNA sequences confirming Splice Patterns	837828	726916
EST/mRNA sequences confirming Transcript Patterns	38731	18045

^1^:AltTrans pipeline requires for a transcript pattern that the underlying splicing and the terminating polyA site are confirmed by the same set of EST./mRNA sequences.

^2^:AltPAS pipeline identifies polyA sites independent of the underlying splicing pattern.

^3^**:**A gene is considered as undergoing alternative polyadenylation if it multiple polyA sites (from the merged list of AltTrans and AltPAS polyA sites) are seen mapped to the gene.

^4^:Consideration of only the AltTrans polyA sites gives a conservative estimate for the extent of alternative polyadenylation. AltTrans polyA sites differ from AltPAS sites in the manner they are detected; AltTrans polyA sites are confirmed by the same set of EST/mRNA sequences that confirm the splice structure of transcript pattern.
